# Characterization of Protective Immune Responses Induced by Pneumococcal Surface Protein A in Fusion with Pneumolysin Derivatives

**DOI:** 10.1371/journal.pone.0059605

**Published:** 2013-03-22

**Authors:** Cibelly Goulart, Thais Raquel da Silva, Dunia Rodriguez, Walter Rodrigo Politano, Luciana C. C. Leite, Michelle Darrieux

**Affiliations:** 1 Centro de Biotecnologia, Instituto Butantan, São Paulo, Brazil; 2 Programa de Pós-Graduação Interunidades em Biotecnologia-USP-IPT-IB, São Paulo, Brazil; 3 Laboratório de Biologia Celular e Molecular, Universidade São Francisco, Bragança Paulista, Brazil; Centers for Disease Control & Prevention, United States of America

## Abstract

Pneumococcal surface protein A (PspA) and Pneumolysin derivatives (Pds) are important vaccine candidates, which can confer protection in different models of pneumococcal infection. Furthermore, the combination of these two proteins was able to increase protection against pneumococcal sepsis in mice. The present study investigated the potential of hybrid proteins generated by genetic fusion of PspA fragments to Pds to increase cross-protection against fatal pneumococcal infection. Pneumolisoids were fused to the N-terminus of clade 1 or clade 2 *pspA* gene fragments. Mouse immunization with the fusion proteins induced high levels of antibodies against PspA and Pds, able to bind to intact pneumococci expressing a homologous PspA with the same intensity as antibodies to rPspA alone or the co-administered proteins. However, when antibody binding to pneumococci with heterologous PspAs was examined, antisera to the PspA-Pds fusion molecules showed stronger antibody binding and C3 deposition than antisera to co-administered proteins. In agreement with these results, antisera against the hybrid proteins were more effective in promoting the phagocytosis of bacteria bearing heterologous PspAs *in vitro*, leading to a significant reduction in the number of bacteria when compared to co-administered proteins. The respective antisera were also capable of neutralizing the lytic activity of Pneumolysin on sheep red blood cells. Finally, mice immunized with fusion proteins were protected against fatal challenge with pneumococcal strains expressing heterologous PspAs. Taken together, the results suggest that PspA-Pd fusion proteins comprise a promising vaccine strategy, able to increase the immune response mediated by cross-reactive antibodies and complement deposition to heterologous strains, and to confer protection against fatal challenge.

## Introduction

*Streptococcus pneumoniae* is a major human pathogen, accounting for over 10% of total deaths in children under the age of five [1]. Despite the well established efficacy of conjugate vaccines against invasive disease, the high production costs involved in the conjugation processes limit their implementation in lower income countries, in which the burden of pneumococcal diseases is highest. Also, due to the limited number of polysaccharides included in the formulations, the extent of vaccine coverage tends to decrease as less prevalent serotypes emerge. In fact, serotype replacement has been observed after the introduction of PCV7 in different populations [2], [3]. Finally, serotype replacement is associated with the emergence of antibiotic resistant clones [4], reinforcing the need for cost-effective strategies that confer broad protection, such as protein-based vaccines.

PspA and Pneumolysin (Ply) are among the most well studied pneumococcal proteins; their contribution to virulence has been demonstrated with mutant strains lacking either one or both proteins, which have shown reduced fitness in different models of colonization, lung infection and bacteremia [5]. Mutant strains were cleared more rapidly from the lungs and blood of mice when compared to wild type counterparts [5], [6] and deposited more C3 *in vitro* [7]. Furthermore, the combination of both mutations had an additive effect on C3 deposition and pneumococcal clearance [6], suggesting that these proteins contribute synergistically to bacterial evasion of innate immune responses [6], [7].

Recombinant forms of PspA and Pneumolysin derivatives (Pds) have been investigated as potential vaccine candidates in different animal models, with promising results. The N-terminal region of PspA, which is responsible for inhibiting complement deposition on the bacterial surface [8], [9] and contains most of the immunogenic epitopes of the molecule [10], confers protection against invasive infection [11]–[13], lobar pneumonia [14] and colonization [15], [16]. Furthermore, it has been recently demonstrated that maternal immunization with PspA protects the offspring against pneumococcal infection [17]. The N-terminus of PspA, however, exhibits structural and serological variability [18]. Based on the observation that different PspA molecules induce antibodies with distinct degrees of cross-reactivity [19], [20] and cross-protection [11], [21], it has been suggested that PspA-based anti-pneumococcal vaccines should include more than one molecule in order to extend coverage. The potential of PspA as a vaccine candidate has been further supported by human clinical trials, which have demonstrated the induction of antibodies with high cross-reactivity against heterologous molecules [21], which can passively protect mice against fatal pneumococcal infection [21].

Pneumolysin (Ply) is a cholesterol dependent cytolysin with several biological effects, such as activation of classical complement pathway [22], induction of apoptosis in numerous cells types [23], [24], impairment of ciliary function in the lungs and induction of oxidative burst by neutrophils [22]. In fact, the instillation of purified Ply in the lungs is sufficient to reproduce many aspects of pneumococcal pneumonia in rats (reviewed in [22]). Furthermore, Ply has been shown to interact with TLR-4 [25] and to induce TLR-4 independent activation of the NLRP3 inflammasome, contributing to host protection against pneumococcal pneumonia [26] and lethal infection [25].

Since Ply is toxic in its native form, several detoxified forms – named pneumolysoids (Pds) – have been produced, by site-directed mutagenesis or chemical detoxification, and evaluated for their immunogenicity and protective effect in different animal models, with variable results, including protection in rhesus macaques [14], [27]–[33]. Of those toxoids, the best characterized are PdB, carrying a Trp-Phe substitution at position 433 [30], and PdT, a triple mutant containing Asp-385 to Asn, Cys-428 to Gly and Trp-433 to Phe substitutions [34]. While PdT alone or co-administered with other pneumococcal antigens, did not induce significant protection against lethal intraperitoneal challenge [29], PdB has been shown to elicit protection against nasal challenge with some pneumococcal strains, which was enhanced by co-administration of other pneumococcal proteins, such as PspA and PhTB [14], [35]. The combination of PspA and PdB elicited the highest protection levels in mouse models of sepsis and focal pneumonia, suggesting a complementary role for these two antigens [14], [36].

On a whole, the results indicate that effective protein-based anti-pneumococcal vaccines tend to require the combination of different proteins in order to extend protection. In the present work, we investigated the ability of fusion proteins including the N-terminal region of family 1 PspAs and detoxified derivatives of Pneumolysin to induce protective immune responses in a mouse model of fatal pneumococcal challenge.

## Materials and Methods

### Pneumococcal Strains

All pneumococcal strains used in this study are shown in Table 1. Pneumococci were maintained as frozen stocks (−80°C) in Todd-Hewitt broth supplemented with 0.5% yeast extract (THY), with 10% glycerol. In each experiment, the isolates were plated on blood agar prior to growth in THY.

**Table 1:** Pneumococcal strains used in this study.

Strain	Serotype	PspA Clades	Source	Reference
P69	10A	1	UFG	20
94/01	18A	2	IAL	20
245/00	14	1	IAL	20
491/00	6B	1	IAL	20
472/96	6B	1	IAL	30
A66.1	3	2	UAB	42
D39	2	2	UAB	5

IAL: Instituto Adolfo Lutz, São Paulo, Brazil.

UFG: Universidade Federal de Goiás, Goiânia, Brazil.

UAB: University of Alabama at Birmingham.

### Cloning of pspAs, pds and Hybrid Genes

**Figure 1:**
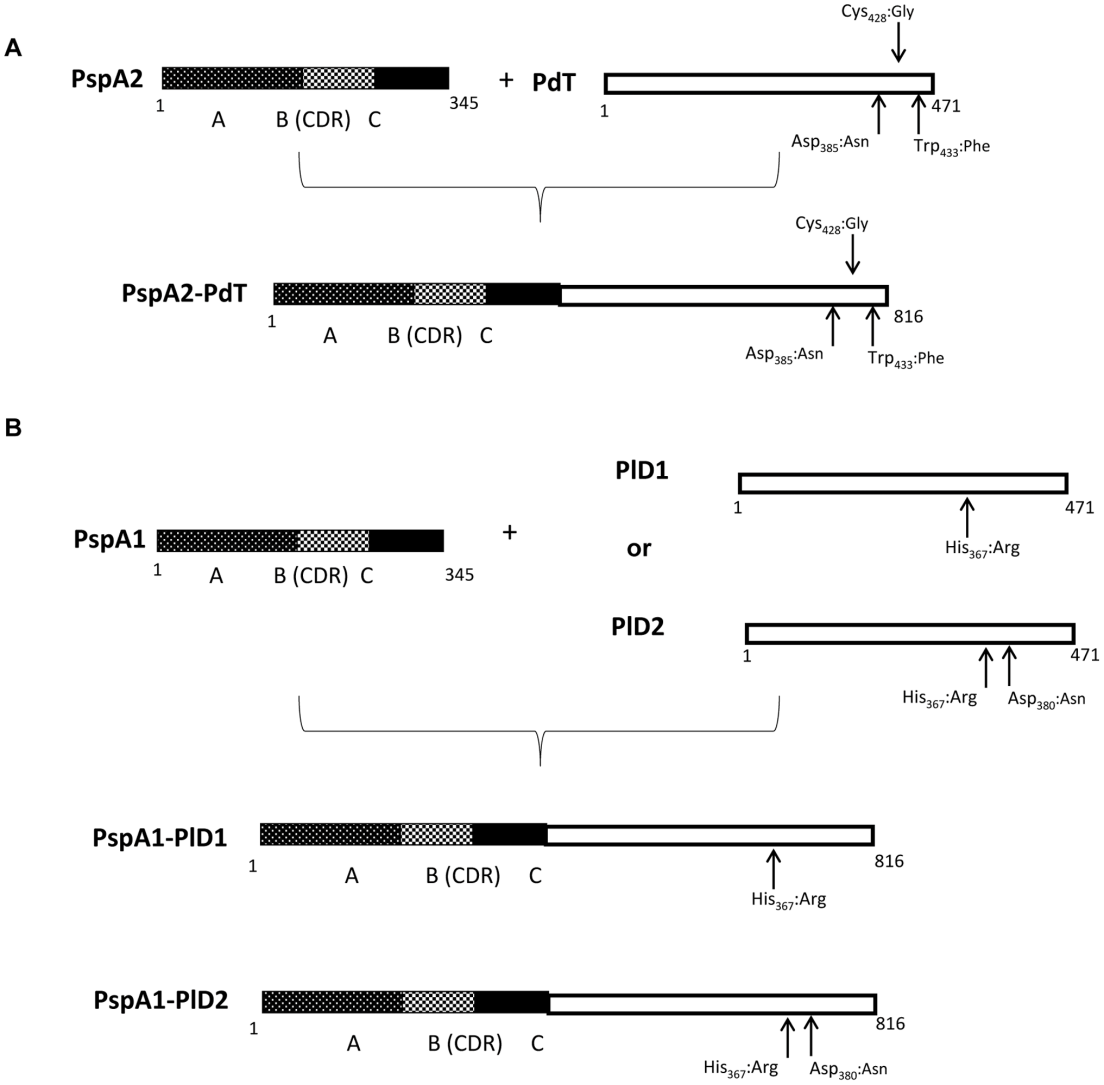
Scheme of Proteins and Hybrids.

Gene fragments encoding the N-terminal region of *pspA* were amplified from pneumococcal strains 245/00 (PspA1) or 94/01 (PspA2) by PCR (Figure 1). The mutant detoxified Pneumolysin gene *pdT* was obtained by PCR from the pQE-30-pdT, kindly provided by Drs. Richard Malley and James Paton. Two more Ply mutants were obtained by site-directed mutagenesis, using the protocol described by Withers-Martinez *et al.* (1999). *Pd_H367_* or *plD1* was amplified from pneumococcal strain D39 and contains one mutation on the His 367 residue, which was substituted by Arg. This mutant was first described by Berry *et al*., 1995, and retains 0.02% of the hemolytic activity of the native protein. The same mutation was inserted in the *ply* gene from strain 472/96, which contains a natural Asp-380 to Asn substitution, in a region described as involved in complement activation (Mitchell *et al.*, 1991). This second mutant, *pD_H367R380_* or *plD2*, contains, therefore, two mutations. The primers used to obtain the mutants are listed in Table S1. The *pspA* fragments and *ply* mutant genes were inserted into pGEM-T easy vector (Promega) and fused through complementary cohesive ends added to the primers, generating three chimeric genes: *pspA1-plD1, pspA1-plD2,* and *pspA2-pdT*. *pspA2-pdT* was digested with the appropriate restriction endonucleases and ligated to the linearized *pQE30* (QIAGEN) expression vector; *pspA1-plD1* and *pspA1-plD2* were excised from pGEM-T easy and subcloned into linearized pAE-6xHis expression vector [37].

### Expression and Purification of Recombinant Proteins

The expression of rPspA2-PdT was performed in *E. coli* M15. The recombinant fragments rPspA1, rPspA2, rPlD1 and rPlD2, as well as the hybrids rPspA1-PlD1 and rPspA1-PlD2 were expressed in *E coli* BL21DE3. All proteins include an N-terminal histidine tag added by the expression vectors *pQE* and *pAE6xHis*. Protein expression was induced in mid-log-phase cultures with 1 mM IPTG (Sigma). rPspA2-PdT, rPspA1 and rPspA2, which were expressed in the soluble form, were purified through affinity chromatography with Ni^2+^ charged chelating Sepharose resin (HisTrap Chelating HP; GE HealthCare) in an Akta Prime apparatus (GE HealthCare), as described by Goulart *et al* [20]. The Pds fragments and hybrids rPspA1-PlD1 and rPspA1-PlD2 were expressed as inclusion bodies; therefore, after cell lysis, the pellets were ressuspended in equilibrium buffer (Tris 50 mM, NaCl 150 mM, Imidazole 5 mM) containing 8 M urea (GE HealthCare) and submitted to refolding by slow dilution in 2 L of equilibrium buffer prior to purification. Elution was carried out with 300 mM imidazole. The purified fractions were analyzed by sodium dodecyl sulfate-polyacrylamide gel electrophoresis (SDS-PAGE), dialyzed against 10 mM Tris-HCl (pH 8), 20 mM NaCl, 0.1% glycine and stored at 20°C.

### Immunoblotting

The expression and purification of the hybrid proteins was confirmed by immunoblotting. Recombinant PspAs or Pds (150 ng of each) and 300 ng of each hybrid protein were separated by SDS-PAGE and transferred to nitrocellulose membranes (GE Healthcare). The membranes containing rPspAs and hybrid proteins or rPds and hybrid proteins were incubated with anti-rPspA or anti-Ply antisera at 1∶4000 and 1∶2000 dilution, respectively, followed by incubation with horseradish peroxidase-conjugated goat anti-mouse IgG (diluted 1∶1000; Sigma). Detection was performed with an ECL kit (GE Healthcare).

### Animals and Immunization

All animal experiments were approved by the Ethics Committee at Instituto Butantan, São Paulo – SP (CEUAIB), (Permit Number: 602/09). Female BALB/c mice from Faculdade de Medicina – Universidade de São Paulo (São Paulo, Brazil) were immunized subcutaneously with 3 doses of 8.8 µg of rPspA2 or rPspA1, 11.2 µg of rPdT, rPlD1 or rPlD2_,_ 20 µg of co-administered proteins (rPspA2+rPdT, rPspA1+rPlD1 *or* rPspA1+rPlD2) or 20 µg of the hybrid proteins at 14-day intervals, using sterile saline solution 0.9% with 50 µg of Al(OH)_3_ as adjuvant (50 µg per mouse). The adjuvant alone in saline was used as a control. Two weeks after the last immunization, the animals were bled by retro-orbital puncture and antibody production was evaluated by ELISA. Serum samples were analyzed individually and comparison among the groups were performed using one-way ANOVA with a Tukey’s Multiple Comparison Test.

### Binding and Complement Deposition Assay

Pneumococcal strains bearing family 1 PspAs (Table 1) were grown in THY up to an optical density of 0.4–0.5 (corresponding to a concentration of 10^8^ CFU/ml) and harvested by centrifugation at 2000×g for 3 min. The pellets were washed once with PBS, ressuspended in the same buffer, and incubated in the presence of heat-inactivated pooled sera from mice immunized with rPspAs, rPds, co-administered proteins or rPspA-Pd fusions at a final concentration of 5% for 30 min at 37°C. The sera were heat-inactivated by incubation at 56°C for 30 min to destroy the activity of serum complement. After washing with PBS, the samples were incubated with 100 µL of PBS containing FITC-conjugated anti-mouse IgG (MP Biomedicals) at 1∶1000 dilution on ice for 30 min in the dark. The bacteria were washed two more times with PBS, ressuspended in 1% formaldehyde and analyzed by flow cytometry, using FACS Canto II (BD Biosciences). For the complement deposition assay, after incubation with antisera, the samples received 10% of BALB/c NMS (normal mouse serum) as a complement source and were incubated at 37°C for another 30 min. The samples were washed two times with PBS and incubated with FITC-conjugated anti-C3 (MP Biomedicals) at a 1∶500 dilution in 100 µL of PBS. The washes, fixation and analysis were performed as previously described [20].

### Opsonophagocytic Assay

The adapted opsonophagocytic assay was performed using pneumococcal strains expressing PspA clades 1 and 2 (Table 1). The bacteria were grown in THY up to mid log phase, harvested by centrifugation at 2000×g for 3 min, washed with PBS and the pellet, ressuspended in opsono buffer [26]. Aliquots containing ∼2.5×10^6^ CFU were incubated with heat inactivated antisera against the recombinant proteins alone, co-administered or the fusion proteins at 1∶16 dilution at 37°C for 30 min. Sera from mice that received saline and Al(OH)_3_ was used as control. After another wash with PBS, the samples were incubated with 10% NMS from BALB/c diluted in opsono buffer at 37°C for 30 min. The samples were then washed once with PBS and incubated with 4×10^5^ peritoneal cells [38] diluted in opsono buffer at 37°C for 30 min with shaking (220 rpm). The reaction was stopped by incubation on ice for 1 min. Ten-fold dilutions of the samples were performed and 10 µL aliquots of each dilution were plated on blood agar plates. The plates were incubated at 37°C, with 5% CO_2_ and the pneumococcal CFU recovered, counted after 18 h. Statistical analysis of the final pneumococcal counts in each group was performed by one-way ANOVA with a Tukey’s Multiple Comparison Test.

### Hemolysis Inhibition Assay

The recombinant Ply was expressed using *E. coli* M15 - RM 86 clone, kindly provided by Drs. Richard Malley and James Paton, and purified by affinity chromatography. The hemolysis inhibition assay was performed in 96 wells plates. 200 µL of sheep whole blood were washed 3 times with PBS and ressuspended in 10 mL of PBS. The antisera produced against PspA2, PdT, PspA2+PdT or PspA2-PdT were incubated with 8 HU (hemolytic units) of Ply at 1∶40 dilution at 37°C for 30 min. The antisera generated by immunization with PspA1, PlD1, PlD2, co-administered proteins or fused proteins PspA1-PlD1 and PspA1-PlD2 were incubated with 4 HU of Ply at 1∶10 dilution at 37°C for 30 min. Serum from mice that received saline and Al(OH)_3_ was used as control. 50 µL aliquots of 2% red blood cells were added at a final concentration of 1%, followed by incubation at 37°C for 30 min. The plates were harvested by centrifugation at 1000×g for 10 min, and the supernatant absorbance was determined at 540 nm.

### Challenge

Two weeks after the last immunization the animals were challenged with 4×10^3^ CFUs of *S. pneumoniae* strain A66.1 or 5×10^6^ CFUs of strain 491/00 injected by the intravenous route. The mice were monitored for 15 days and the differences between the survival rates in each group were analyzed by Mann–Whitney U test. Morimbund mice or animals that developed paralysis were euthanized by CO_2_ narcosis. At the endpoint, all surviving animals were euthanized.

## Results

### Expression and Purification of rPspA-Pd Hybrid Proteins

**Figure 2:**
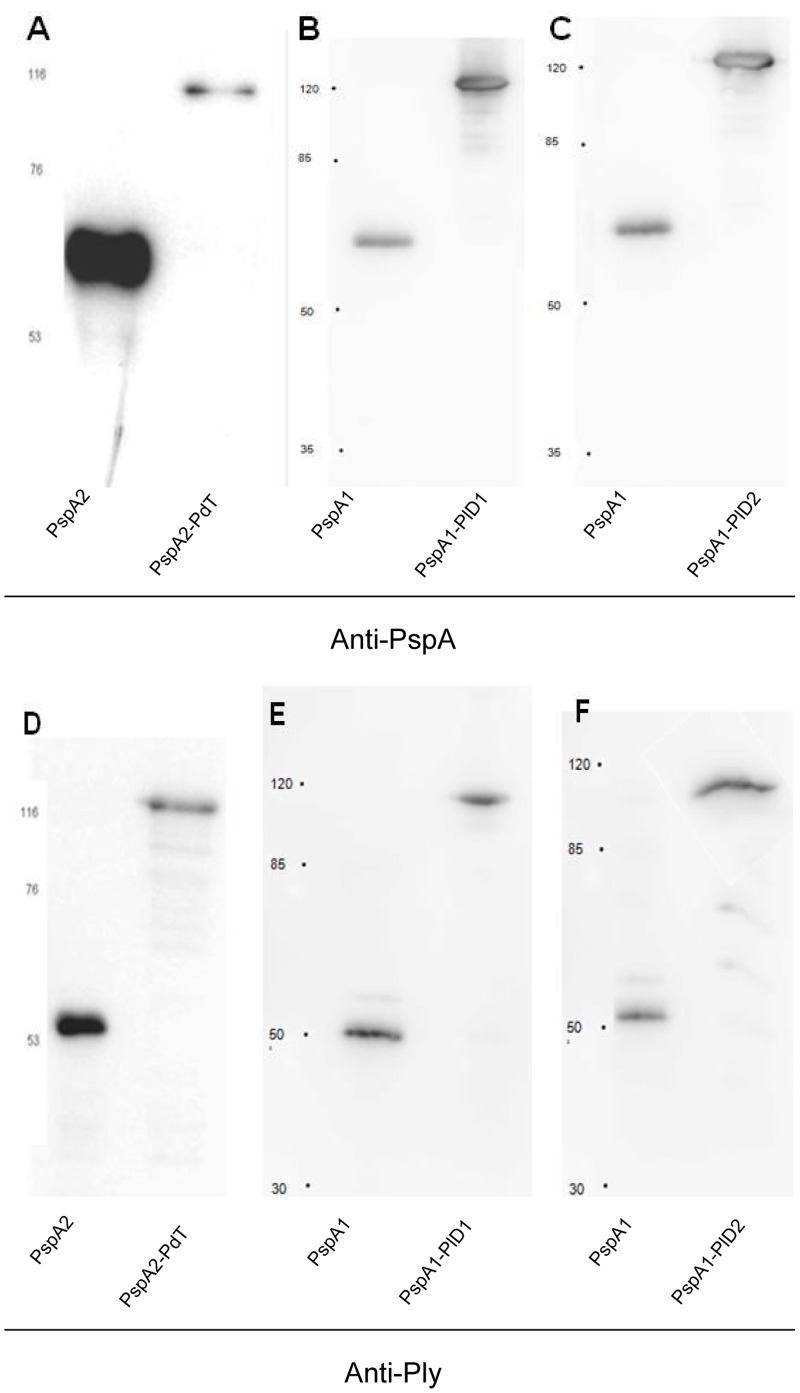
Recognition of hybrid proteins by antibodies against PspA and Pds. Recombinant proteins were separated by SDS-PAGE and transferred to PVDF membrane - A) rPspA2 and rPspA2-PdT; B) rPspA1 and rPspA1-PlD1; C) rPspA1 and rPspA1-PlD2; D) rPdT and rPspA2-PdT; E) rPlD1 and rPspA1-PlD1; and F) rPlD2 and rPspA1-PlD2. The membranes were incubated with anti-rPspA2 (A) anti-rPspA1 (B and C) or anti-rPly (D, E and F) followed by incubation with anti-mouse IgG conjugated with HRP. Detection was performed with an ECL kit (GE Healthcare). Molecular mass markers (kDa) are indicated on the left.

The gene fragments encoding rPspAs and rPds were fused with restriction enzymes and expressed in *E. coli* using vectors that add a Histidine tag to the beginning of the aminoacid sequence. Both pspA gene fragments contain the N-terminal region including the proline rich region and the non-proline block; the scheme of proteins and hybrids is shown in Figure 1.The rPspA1-PlD1 and rPspA1-PlD2 hybrids were expressed in inclusion bodies, denatured with urea and refolded. rPspA2-PdT was expressed in soluble form. All proteins were purified through Ni^2+^-affinity chromatography and analyzed by immunoblotting using anti-rPspA1 or anti-rPspA2 and anti-rPly antibodies (Figure 2). All hybrid proteins were expressed and purified integrally and were recognized by antibodies against PspA or Ply individually.

### Mouse Immunization with Hybrid Proteins Induce Levels of Antibodies Comparable to Proteins Administered Individually

Sera obtained by BALB/c immunization with rPspAs, rPds, and rPspA-Pds were quantified by ELISA against each recombinant protein. Immunization with the hybrid proteins induced antibody levels comparable to those obtained in the groups immunized with each antigen alone (Table 2).

**Table 2:** Antibody levels in mice immunized with the recombinant proteins.

Coating antigen	Antisera	Antibodies concentration (µg/mL)	*P value*
PspA 2	PspA2	13,350	<0,01
	PspA2-PdT	13,740	<0,01
PdT	PdT	91	<0,05
	PspA2-PdT	126	<0,01
PspA1	PspA1	10,140	<0,001
	PspA1-PlD1	12,530	<0,001
	PspA1-PlD2	7,000	<0,01
PlD1	PlD1	245	<0,05
	PspA1-PlD1	313	<0,01
PlD2	PlD2	167	<0,05
	PspA1-PlD2	241	<0,01

* *p* values were calculated in comparison with the control group.

### Antibodies Generated by Mouse Immunization with Hybrid Proteins Bind to the Surface of Pneumococci Bearing Different PspAs

**Figure 3:**
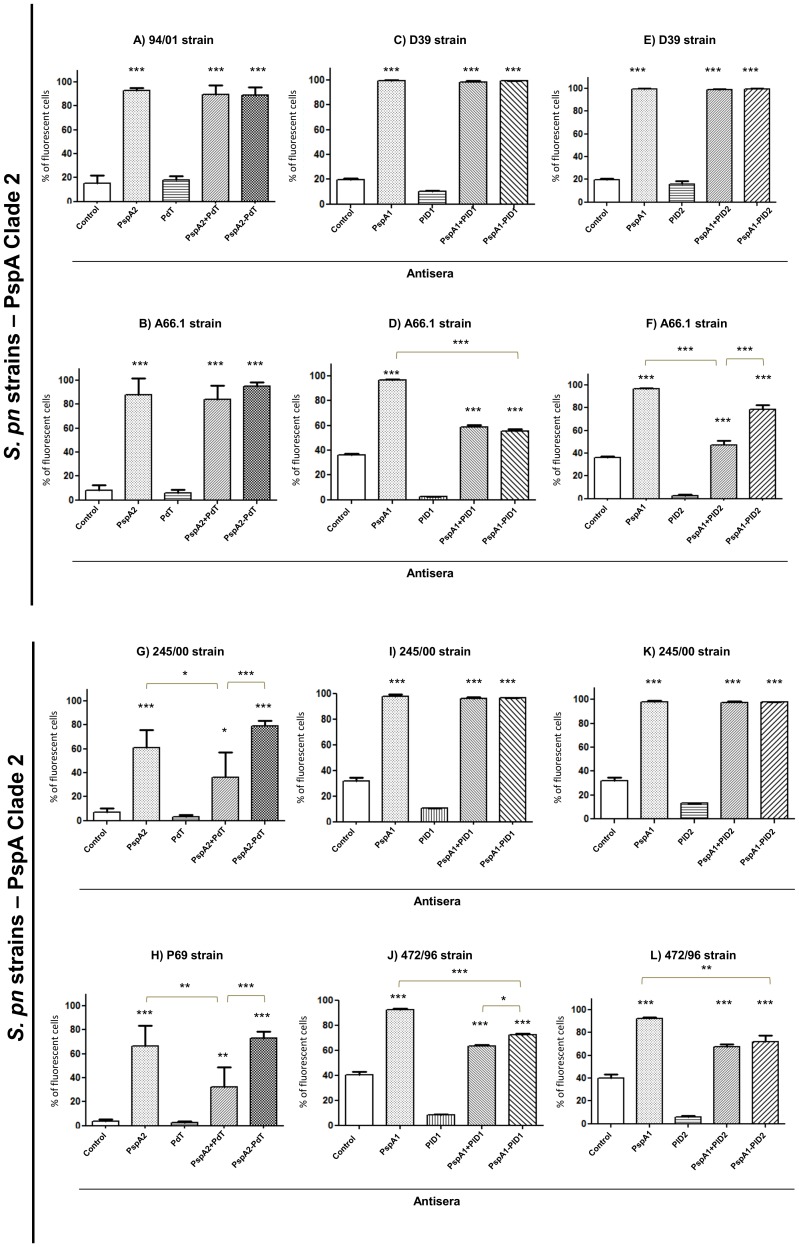
Antibody binding onto pneumococcal surface. Pneumococcal strains containing PspA2 or PspA1 were incubated with antisera from mice immunized with rPspAs, rPds, co-administered proteins or hybrids, followed by incubation with anti-IgG mouse conjugated with FITC and analyzed by FACS. Serum from mice that received saline/Al(OH)_3_ was used as a control. The percentage of fluorescent bacteria (>10 fluorescence intensity units) was calculated for each sample. Statistical analysis was performed by one-way ANOVA with a Tukey’s Multiple Comparison Test : *p<0.05; **p<0.01; ***p<0.001 for treated versus control or between immunized groups, as indicated.

Sera from mice immunized with the recombinant proteins and hybrids were tested for their ability to bind onto the pneumococcal surface. Pneumococci bearing family 1 PspAs were incubated with the antisera followed by incubation with anti-mouse IgG-FITC. Antibodies generated against rPspA2-PdT were able to bind to the surface of pneumococci bearing homologous PspAs (clade 2), similarly to anti-rPspA2 or anti-rPspA2+PdT antisera (Figure 3–A and B). Interestingly, this same antiserum was able to bind with significantly higher affinity to pneumococcal strains bearing heterologous clade 1 PspAs when compared with antiserum generated against the co-administered proteins (Figure 3 G and H). Antisera generated against the other hybrids, rPspA1-PlD1 and rPspA1-PlD2, also revealed strong binding to pneumococcal strains, usually comparable to that observed with antisera against the co-administered proteins (Figures 3 C, E, D, I, K and L), with few exceptions (Figure 3 F and J). However, in some cases, the hybrid proteins showed a binding capacity lower than that observed for anti-PspA antisera alone (Figures 3 D, J and L). No binding was observed when anti-Pds antisera were used.

### Antibodies Induced against rPspA-Pd Hybrids Induced an Increased C3 Deposition on Pneumococci Bearing Heterologous PspAs

**Figure 4:**
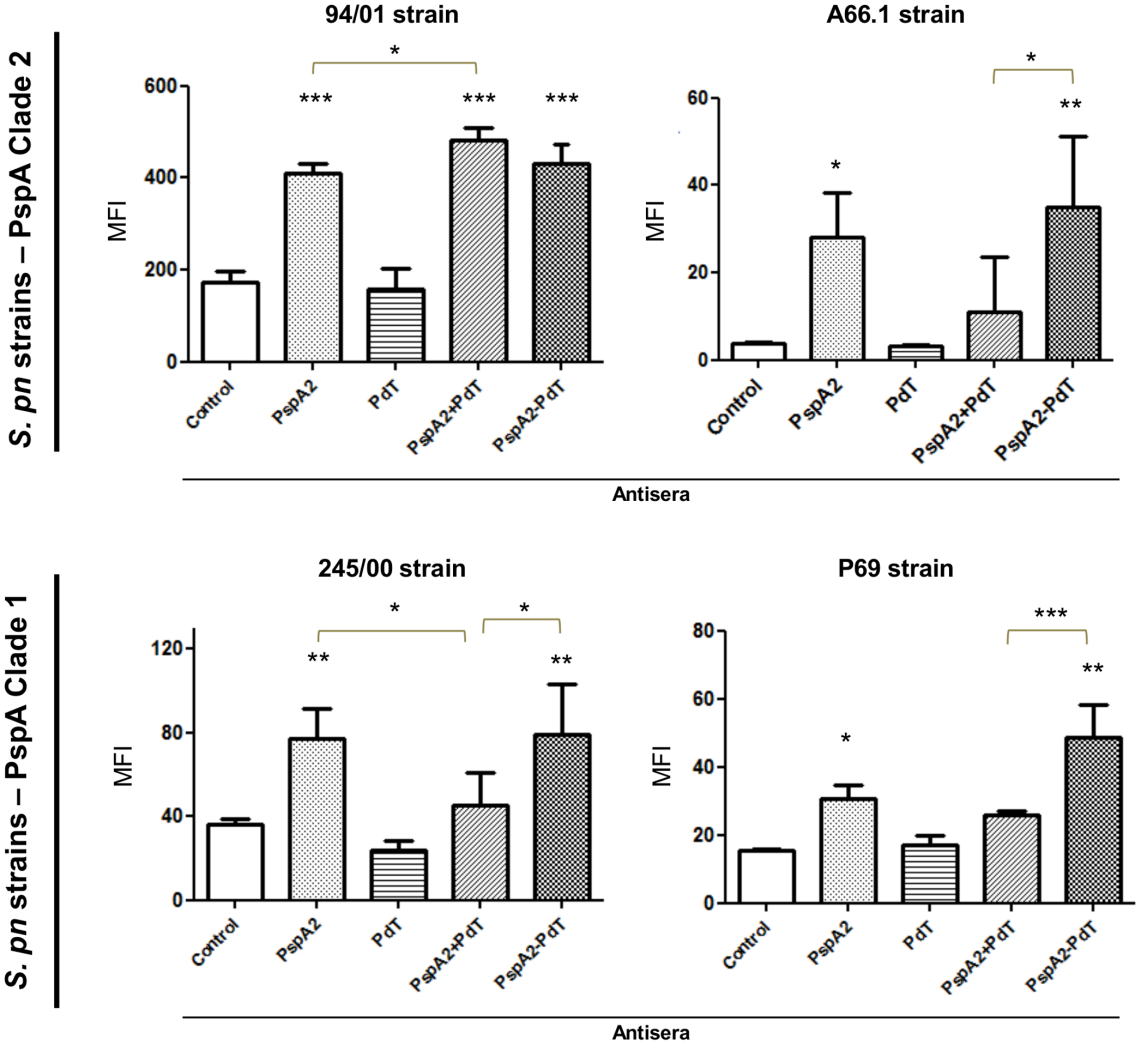
Complement deposition on pneumococcal surface in the presence of specific antibodies. Pneumococcal strains were incubated with antisera from mice immunized with rPspAs, rPds, co-administered proteins or hybrids and NMS as complement source. After incubation with anti-C3 mouse conjugated with FITC, the samples were analyzed by FACS. Serum from mice that received saline/Al(OH)_3_ was used as a control. The median of fluorescence intensity (MFI) was calculated for each sample. Statistical analysis was performed by one-way ANOVA with a Tukey’s Multiple Comparison Test: *p<0.05; **p<0.01; ***p<0.001 for treated versus control or between immunized groups, as indicated.

The antisera were evaluated as to their ability to increase complement deposition on the surface of pneumococci. *S. pneumoniae* strains expressing PspA clade 1 or clade 2 were incubated with the antisera generated against the rPspA-Pd hybrids and respective controls, in the presence of a complement source, followed by incubation with anti-C3 conjugated with FITC, and these were analyzed by FACS. When we used a bacterium containing a homologous PspA, we observed that antibodies from mice immunized with co-administered proteins showed increased complement deposition when compared with anti-serum from the group immunized with rPspA alone (Figure 4–94/00 strain). However, comparable levels of C3 deposition were observed when the same bacteria was incubated with serum from the group immunized with rPspA2-PdT or the antisera induced against the co-administered proteins, which is in agreement with the results of antibody binding. On the other hand, the anti-rPspA2-PdT antibodies induced significantly higher amounts of C3 deposition on the bacterial surface, as compared to antibodies induced against the co-administered proteins for all the other strains used in this study, including those bearing PspA2 or PspA1 (Figure 4 A66.1, 245/00, P69 strains). In relation to the antisera induced against the rPspA1-PlD1 and rPspA1-PlD2 hybrids, no significant differences were observed in C3 deposition on pneumococci when compared with antisera generated against rPspA1 alone or the co-administered proteins, also in accordance with antibody binding (Figure S1).

### In-vitro Opsonophagocytosis Mediated by Anti-hybrid Antisera is More Efficient than Antisera against Co-administered Proteins in Heterologous Strains

**Figure 5:**
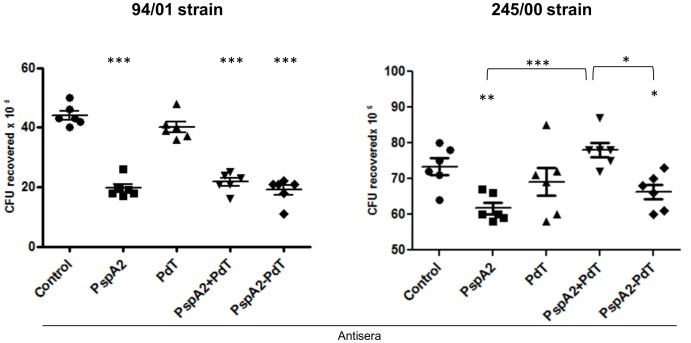
Pneumococcal phagocytosis mediated by specific antibodies in the presence of complement. Pneumococcal strains bearing PspA2 (94/01 strain) or PspA1 (245/00 strain) were incubated with antisera from mice immunized with rPspA2, rPdT, rPspA2+rPdT or rPspA2-PdT and NMS as complement source, followed by incubation with mouse peritoneal phagocytes and plated on blood agar plates. CFU recovered were counted after 18 h. Statistical analysis was performed by one-way ANOVA with a Tukey’s Multiple Comparison Test. *p<0.05; **p<0.01; ***p<0.001 for treated versus control or between immunized groups, as indicated.

Since the immunization with the rPspA2-PdT hybrid induces antibodies that exhibited both a stronger binding capacity and a more pronounced C3 deposition on the bacterial surface when compared to the other groups, these antibodies were investigated for their ability to mediate the opsonophagocytosis and killing of pneumococci *in vitro*. Bacterial strains bearing PspA clade 1 or clade 2 were incubated with antisera against rPspA2, rPdT, rPspA2+rPdT and rPspA2-PdT and a complement source, followed by incubation with murine peritoneal phagocytic cells. The samples were plated and the number of CFUs recovered after 18 h were counted. When we used the pneumococcal strain 94/00, expressing the homologous PspA clade 2, antibodies generated against the hybrid, rPspA2-PdT, promoted a significant reduction in the number of CFU recovered when compared to control or anti-rPdT anti-serum (Figure 5). This reduction was similar to that observed when incubating with anti-rPspA2 or the sera from the co-administered antigens, anti-rPspA2+rPdT (Figure 5–94/00 strain). However, when a pneumococcal strain bearing a heterologous PspA molecule (clade 1) was used, the anti-rPspA2-PdT anti-serum was more efficient in promoting the opsonophagocytic killing of the bacterium than antisera against the co-administered proteins (Figure 5–245/00 strain).

### Inhibition of Ply Cytolytic Activity by Anti-pneumolysoids Antibodies

**Figure 6:**
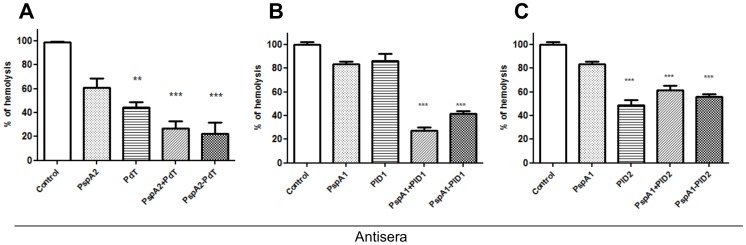
Inhibition of hemolytic activity of Ply on red blood cells by antisera generated against recombinant and hybrids proteins. Ply was incubated with antisera and sheep red blood cells, and the supernatant absorbance was measured at 540 nm. Results are shown as percentages of the hemolytic activity in presence of sera from mice receiving saline and Al(OH)_3_ (control). Statistical analysis was performed by one-way ANOVA with a Tukey’s Multiple Comparison Test.; **p<0.01; ***p<0.001.

The ability of antibodies generated against PspA1, PspA2, pneumolysoids, co-administered proteins or fused proteins to inhibit the lytic effects of Pneumolysin was tested by incubation with sheep red blood cells in the presence of recombinant Ply. All antisera from formulations including pneumolysoids significantly reduced the hemolysis of red blood cells by Pneumolysin, with the exception of anti-PlD1 (Figure 6). As expected, antibodies to PspA1 and 2 did not induce a significant inhibition of hemolysis (Figure 6).

### Immunization with PspA-Pds Leads to an Increased Survival against Fatal Pneumococcal Challenge

**Figure 7:**
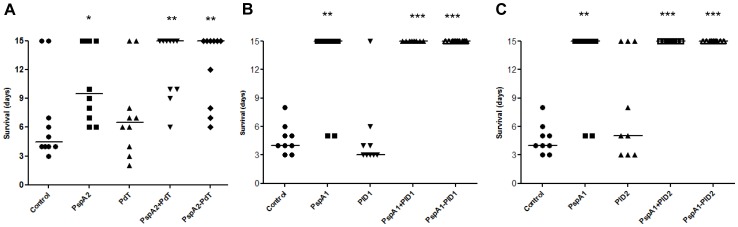
Mouse immunization with rPspA-Pds confers protection against pneumococcal sepsis with strains bearing heterologous PspAs. BALB/c mice immunized with 3 doses of rPspAs, rPds, co-administered or hybrid proteins were challenged with lethal doses of pneumococcal strains bearing heterologous PspA: A – Mice were challenged with 491/00 strain (PspA1); B and C – Mice were challenged with A66.1 strain (PspA2). The mice were monitored for 15 days and differences between the survival rates in each group were analyzed by Mann–Whitney U test, *p<0.05; **p<0.01; ***p<0.001 for treated versus control groups.

The protective effect of rPspA-Pd fusions was evaluated in comparison with the isolated rPspA, rPds or the co-administered proteins, by intravenous lethal challenge with the virulent pneumococcal strains St 491/00 (Figure 7 - A) or A66.1 (Figure 7 - B and C), expressing PspA clades 1 or 2, respectively. Figure 6 shows the survival of mice up to 15 days after challenge, when the experiment was terminated. All rPspA-Pd hybrids induced higher survival rates in comparison with the control group or Pds. Furthermore, the animals immunized with the hybrids showed an increased, but not significant, survival when compared to those immunized with rPspA alone. In fact, two of the hybrids, rPspA1-PlD1 and rPspA1-PlD2 induced 100% protection against pneumococcal challenge. No differences in protection were observed between mice receiving the hybrids or co-administered proteins. Immunization with rPds alone, on the other hand, did not induce protection in this model of systemic infection.

## Discussion

PspA and Pds have been studied as vaccine candidates against pneumococcal infections for decades, with striking success in different animal models and, in the case of rPspA, induction of antibodies with high cross-reactivity [21] and protective potential in humans [21].

Since rPspA exhibits structural and serological variability [18], it has been suggested that the inclusion of two or more fragments would be necessary in order to increase vaccine coverage. Fusion of PspA fragments of families 1 and 2 have been demonstrated to increase protection against invasive pneumococcal infection [13], [39], as well as rPspA fusion or co-administration with adjuvant molecules [40], [41]. Nevertheless, the width of protection could be further extended by including a more conserved protein in the formulation. Several formulations containing more than one pneumococcal protein have been studied [14], [33], [42]–[44]. Furthermore, it has been suggested that vaccines including the same components as mixtures or fused formulations can differ in the levels of protection that they induce. A study from Lu *et al.* [42] demonstrated protection against fatal pneumococcal infection in mice immunized with trivalent vaccine containing fusions of rPdT, rPsaA and cell wall polysaccharide, but not with the antigens mixture. The same was observed using rPspA fused or mixed with flagellin, as an adjuvant molecule [40], suggesting that fused antigens could be more effective than co-administered formulations.

The present work investigated the potential of rPspA-Pd fusions to protect mice against invasive pneumococcal infections. Three fusions were produced, including two rPspA fragments and three different pneumolysoids. These fusion proteins were recognized by antibodies made against both PspA and the Pneumolysoids by Western blotting, showing that both proteins in the constructs were expressed and purified in the integral form, allowing for specific antibody recognition. The antibody levels were measured by ELISA against each recombinant protein. Although PspA was more immunogenic than the Pds (inducing antibody levels around a thousand times higher), immunization with the hybrids induced antibody levels comparable to those produced in mice immunized with each antigen alone, indicating that no antigenic competition occurred in the fusion.

Antibodies against the recombinant proteins and hybrids were evaluated for the ability to recognize and bind to intact pneumococci by FACS. Sera from mice immunized with the fusions revealed a strong binding capacity to all pneumococci tested, comparable – and in some cases superior – to that of sera against the co-administered proteins. Particularly, sera from mice immunized with rPspA2-PdT showed a stronger binding capacity to a PspA clade 1 bearing strain, when compared to the co-administered proteins. The correlation between sequence identity among PspAs and antibody binding has been demonstrated in other studies, with variable results [11], [13], [19], [20]. In general, a strong association between PspA type and the ability of induced antibodies to recognize the PspA molecules in the bacterial surface has been observed. Therefore, the significant increase in recognition of a PspA clade 1 strain by antibodies against a clade 2-containing hybrid suggests that the genetic fusion of PspA and Pd, may have a positive effect on the immune response induced against PspA.

Recent studies using cell fractionation and Western-blotting have demonstrated that Ply localizes in the pneumococcal cell wall compartment [45], [46]. However, the absence of antibody recognition observed with antibodies to Pds alone suggests that this protein is not accessible to antibodies in intact pneumococci. These results were in accordance with previous studies, which suggest that Ply is not displayed on the pneumococcal surface [47], [48]. Since antibodies to Pds alone do not interact with the bacterial surface, the increased binding capacity of anti-hybrid antiserum onto pneumococci bearing hetelogous PspAs when compared with anti-rPspA antiserum could be explained by a possible modification in the PspA structure caused by PdT fusion, that promoted the presentation of more conserved epitopes or by an adjuvant effect of Ply [49].

Complement deposition is the key for opsonization and phagocytosis of pneumococci. Therefore, since PspA and Ply have both been shown to interact with complement components, we investigated the ability of the induced antibodies to promote C3 deposition onto the bacterial surface. In agreement with the binding results, antibodies to the rPspA2-PdT fusion protein mediated a significant enhancement in the levels of C3 deposited on the bacterial surface in relation to antibodies generated against co-administered proteins.

In order to investigate whether the produced antibodies were able to mediate the opsonophagocytosis and killing of pneumococci, bacteria were incubated in the presence of sera induced against either the recombinant proteins alone, co-administered, or the fusion proteins, and mouse peritoneal phagocytes. Corroborating with the C3 complement deposition results, serum induced against the rPspA2-PdT fusion protein was able to promote the opsonophagocytosis and killing of pneumococcal strains. Furthermore, when compared with antisera induced against the co-administered proteins, the anti-hybrid antiserum showed a significantly increased ability to reduce the number of CFU recovered in a strain containing a heterologous PspA.

In accordance with the in vitro assays, which revealed a strong ability of antibodies against the hybrids to mediate complement deposition and phagocytic killing of bacteria, immunization with rPspA-Pd fusions protected mice against fatal challenge with pneumococcal strains bearing heterologous PspA molecules. The results also provide an insight on the mechanism responsible for protection in this model, with the induction of antibodies capable of enhancing C3 deposition on the bacterial surface, which in turn become more susceptible to phagocytic killing.

Quin et al (2007), using mutant Ply-negative pneumococci, observed that deletion of Ply did not affect blood clearance in comparison with a wild type strain. Therefore, we did not expect the Pds alone to be protective against an intravenous challenge. In fact, none of the Pds tested confer protection in this model of infection. However, anti-Pd antibodies could neutralize the lytic effects of Ply. The ability of such antibodies to mediate protective responses was evaluated through a hemolysis inhibition assay using sheep red blood cells. All formulations including Pds induced antibodies able to inhibit hemolysis by Ply, except PlD1. This result indicates that antibodies against Pds may play a role in protection from *Streptococcus pneumoniae* infections by inhibiting Ply’s lytic effects. The correlation between the capacity to inhibit Ply induced hemolysis and protection conferred by Pds has been confirmed by Salha et al (2012), using a detoxified mutant PlyD1 [32].

Taken together, the results suggest that PspA-Pd fusion proteins comprise a promising vaccine strategy, able to increase the immune response mediated by cross-reactive antibodies and complement deposition to heterologous strains, and to confer protection against fatal challenge.

## Supporting Information

Figure S1 Complement deposition on pneumococcal surface in the presence of specific antibodies. Pneumococcal strain D39 was incubated with antisera from mice immunized with rPspA1, rPlD1, co-administered proteins or PspA1-PlD1 hybrid (A), or rPspA1, PlD2, co-administered proteins or PspA1-PlD2 hybrid (B) and NMS as complement source. After incubation with anti-C3 mouse conjugated with FITC, the samples were analyzed by FACS. Serum from mice that received saline/Al(OH)_3_ was used as a control. The median of fluorescence intensity (MFI) is shown for each sample.
(TIF)

Table S1 Oligonucleotides used in this study: sequence of the primers used for amplification of the PspA fragments and for insertion of the mutation on the Ply gene is shown.
(DOCX)
