# Supplementary figures and images for: Characterization of Protective Immune Responses Induced by Pneumococcal Surface Protein A in Fusion with Pneumolysin Derivatives
Source: PLoS One. 2013 Mar 22;8(3):e59605. doi: 10.1371/journal.pone.0059605 (PMC3606166; doi:10.1371/journal.pone.0059605)

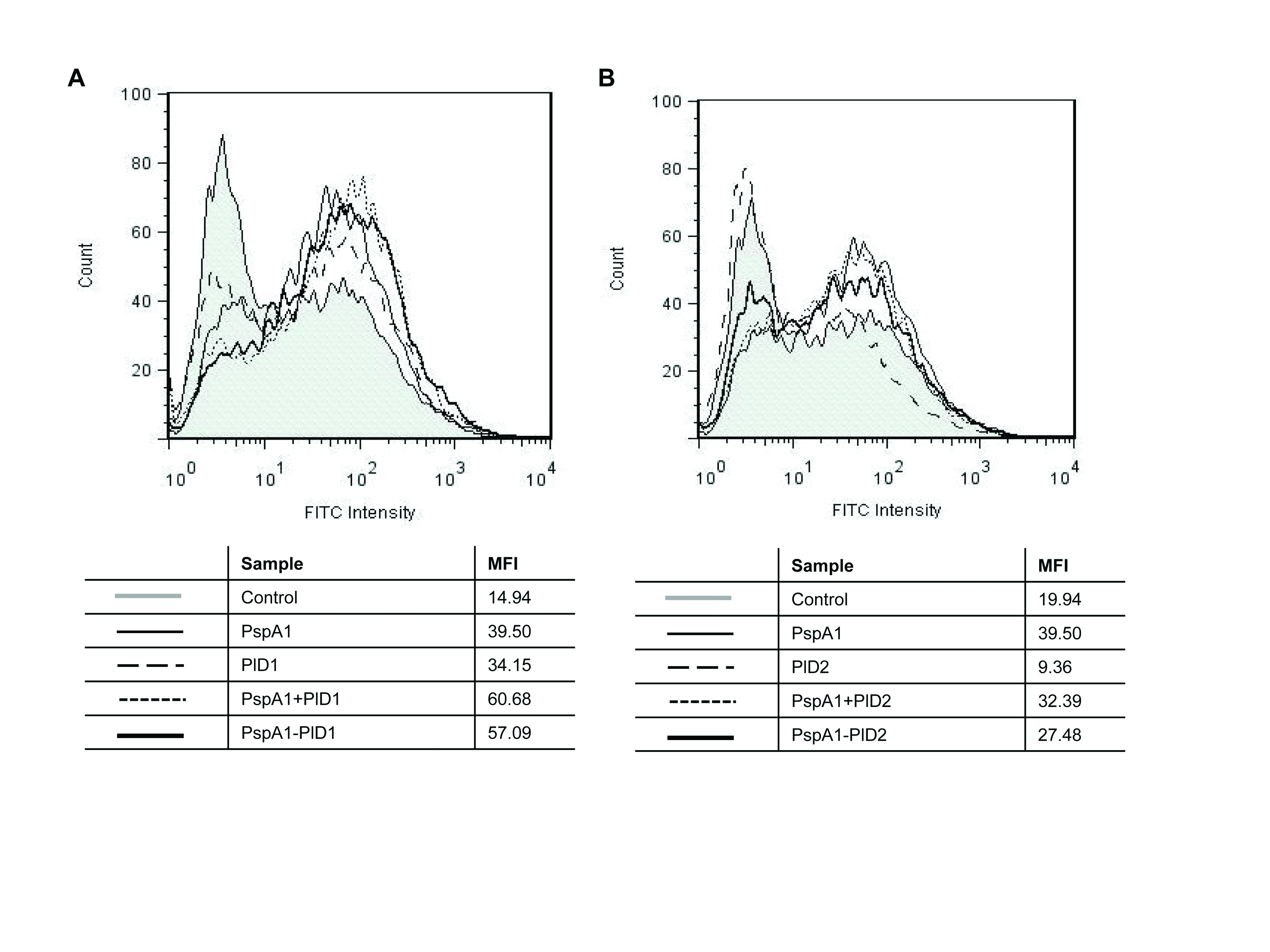

Supplement: Figure S1 — Complement deposition on pneumococcal surface in the presence of specific antibodies. Pneumococcal strain D39 was incubated with antisera from mice immunized with rPspA1, rPlD1, co-administered proteins or PspA1-PlD1 hybrid (A), or rPspA1, PlD2, co-administered proteins or PspA1-PlD2 hybrid (B) and NMS as complement source. After incubation with anti-C3 mouse conjugated with FITC, the samples were analyzed by FACS. Serum from mice that received saline/Al(OH)3 was used as a control. The median of fluorescence intensity (MFI) is shown for each sample. (TIF) [file pone.0059605.s001.tif]
