# Supplementary material for: Characterization of Protective Immune Responses Induced by Pneumococcal Surface Protein A in Fusion with Pneumolysin Derivatives
Source: PLoS One. 2013 Mar 22;8(3):e59605. doi: 10.1371/journal.pone.0059605 (PMC3606166; doi:10.1371/journal.pone.0059605)
Supplement: Table S1 — Oligonucleotides used in this study: sequence of the primers used for amplification of the PspA fragments and for insertion of the mutation on the Ply gene is shown. (DOCX) [file pone.0059605.s002.docx]

Table S1. Oligonucleotides used in this study

| Application | Sequence |
| --- | --- |
| PspA – Forward | 5’ – GAAGCGCCCGTAGCTAGTC – 3’ |
| PspA – Reverse | 5’ – TTCTGCTTTTGGCGGTTGCTG – 3’ |
| Ply - Forward | 5’ – GCA AAT AAA GCA GTA AAT GAC TT – 3’ |
| Ply – Reverse | 5’ – ATT TTC TAC CTT ATC CTC – 3’ |
| Ply_H367R_ – Mutation Forward* | 5’ – CTG GAT C**G**T AGT GGT GCC – 3’ |
| Ply_H367R_ – Mutation reverse* | 5’ – GGC ACC ACT A**C**G ATC CAG – 3’ |

*These are internal primers which overlap on the point where the H367R mutation was inserted (underlined bases).
